# Available and unavailable decoys in capuchin monkeys (*Sapajus* spp.) decision-making

**DOI:** 10.1007/s10071-024-01860-y

**Published:** 2024-02-23

**Authors:** Marco Marini, Edoardo Colaiuda, Serena Gastaldi, Elsa Addessi, Fabio Paglieri

**Affiliations:** 1 IMT School for Advanced Studies, Lucca, Italy; 2 Institute of Cognitive Sciences and Technologies, National Research Council, Rome, Italy

**Keywords:** Context effects, Attraction effect, Unavailable decoys, Phantom decoy, Capuchin monkeys, Comparative decision-making

## Abstract

Decision-making has been observed to be systematically affected by decoys, i.e., options that should be irrelevant, either because unavailable or because manifestly inferior to other alternatives, and yet shift preferences towards their target. Decoy effects have been extensively studied both in humans and in several other species; however, evidence in non-human primates remains scant and inconclusive. To address this gap, this study investigates how choices in capuchin monkeys (*Sapajus spp.*) are affected by different types of decoys: asymmetrically dominated decoys, i.e., available and unavailable options that are inferior to only one of the other alternatives, and phantom decoys, i.e., unavailable options that are superior to another available alternative. After controlling for the subjective strength of initial preferences and the distance of each decoy from its target in attribute space, results demonstrate a systematic shift in capuchins’ preference towards the target of both asymmetrically dominated decoys (whether they are available or not) and phantom decoys, regardless of what options is being targeted by such decoys. This provides the most comprehensive evidence to date of decoy effects in non-human primates, with important theoretical and methodological implications for future comparative studies on context effects in decision-making.

**Supplementary Information:**

The online version contains supplementary material available at 10.1007/s10071-024-01860-y.

## Introduction

Traditional economic theories of rational decision-making assume that preferences are consistent and context-independent. Consistency implies that the relative preferences among two options should not be reversed if a third alternative is added to the choice set. Similarly, context independence imposes that the evaluation of an option should not be affected by the presence of an inferior, irrelevant, or unobtainable alternative (for a review, see Rieskamp et al. 2006). Whatever their merits as a normative model of rational choice, these economic axioms failed the empirical test. Decision makers' preferences are sensitive to the context and change according to the quantity and quality of the available alternatives (Lichtenstein and Slovic 2006). Context effects prove that manipulating the choice set by adding irrelevant alternatives shifts the preference order and changes the attribution of subjective values (Pettibone and Wedell 2000; Prelec et al. 1997).

The primary objective of this study is to investigate the presence of context effects in capuchin monkeys, shedding light on whether these cognitive biases are shared across species and, if so, their underlying mechanisms. By bridging the gap between human and non-human animal decision-making, this research contributes valuable insights into the evolution and universality of context effects, with potential implications for understanding decision-making processes in a broader context.

### Real and phantom decoys

One of the most studied context effects is the attraction effect (AE; Huber et al. 1982; also known as the decoy effect or asymmetric dominance effect). It shows that adding to a binary choice set a new alternative (decoy), that is built to be clearly inferior to one of the pre-existing options, increases the relative choice share of the dominating alternative (target), at the expense of the other option (competitor; Huber et al. 1982). For example, the probability of choosing a print-and-web subscription to a journal that costs 125$ compared to the online subscription at 59$ is higher when the choice set also includes the only print subscription at 125$, a clearly irrelevant and inferior option (Ariely 2008). To elicit AE, alternatives are usually described on at least two attribute dimensions (i.e., quality and price), thus eliciting so-called “multiattribute decision making”; moreover, the target alternative must clearly dominate the decoy option, which in turn must not be clearly dominated by the competitor option. Most of the previous literature employed decoys built to be equally rewarding on one dimension (i.e., quality) but evidently disadvantageous on the other attribute (i.e., price) compared to the dominant target option (“asymmetrically dominated decoys”—ADD; Lichters et al. 2015).

To date, context effects have been repeatedly observed in humans, both in laboratory studies and real-life scenarios, across various domains. To mention a few: political elections (Herne 1997), risky and intertemporal decisions (Marini and Paglieri 2019), consumer choices (Huber et al. 1982), medical and legal judgments (Schwartz and Chapman 1999; Kelman et al. 1996), dating preferences (Ariely, 2008), and many others. Importantly, in recent years, context effects have also been demonstrated in merely perceptual tasks (Trueblood et al. 2013; Liao et al. 2021), where decisions require a simple perceptual discrimination (i.e., choosing between stimuli based on their physical characteristics, namely width or height), in contrast with preferential-based decisions, that are instead managed by high-level cognitive processes (Busemeyer et al. 2019). Since perceptual choice tasks entail quick and intuitive decisions that do not need deliberation or justifications, it is reasonable to assume that context effects would have emerged early in ontogenetic and phylogenetic development (Zhen and Yu 2016). Integrated models of the role of context effects on, respectively, perceptual and value-based choices have also been proposed: for instance, a recent process tracing study suggests that context effects may, at first, be elicited by perceptual cues of the choice set (i.e., attribute salience), before being strengthened by higher cognitive operations (such as value integration and attribute-wise comparative processes; Marini et al. 2020, 2022).

Interestingly, another, markedly different, type of decoy can also affect the decision-making process: unavailable decoys (PDs; Pettibone and Wedell 2007). A phantom decoy is an option inserted into the choice set that can be compared with the others and evaluated against them, yet it remains unavailable for actual selection. Contrary to asymmetrically dominated decoys, phantom decoys typically dominate the target option; some studies also use inferior phantom decoys (e.g., Doyle et al. 1999; Wu and Yu 2020), but this practice is much less widespread than adopting superior phantom decoys. Despite its unavailability, the unobtainable option increases preferences for the asymmetrically dominated target, which gains a significant share when a superior unavailable alternative (the phantom decoy) is presented jointly with the choice set (Pettibone and Wedell 2007). Phantom superior unobtainable decoys, first introduced by Pratkanis and Farquhar (1992) in opposition to real decoys (available inferior options), have been subsequently divided into known and unknown phantoms (Scarpi and Pizzi 2013). Known PDs are clearly labeled as unavailable (i.e., sold-out products) from the stimulus onset, while unknown PDs seem to be authentic available options until the decision maker tries to select them. Whereas known PDs usually strengthen target preferences, unknown PDs trigger a reactance process that leads to higher competitor selections (Scarpi and Pizzi 2013).

### Theories of context effect

Several explanations of these context effects have been proposed. With respect to ADDs, available theories include weight-based explanations (Huber et al. 1982), prospect theory (Kahneman 1979), high-level cognitive explanations (Simonson 1989; Hedgcock and Rao 2009), perceptual explanations (Dimara et al. 2017), and salience-driven processes (Bordalo et al. 2013). As regards PDs, the two main theories that have been used to explain its elicitation are the relative advantage model (Tversky and Simonson 1993) and the similarity-substitution hypothesis (Pettibone and Wedell 2000). Tversky and Simonson (1993) suggested that the phantom option serves as the reference point during decision-making, with only the relative advantages and disadvantages of the alternatives being considered. In contrast, Pettibone and Wedell (2000) suggest that decision-makers, using a heuristic strategy, tend to select the option most similar to the unavailable one without reordering their preferences. However, given the pervasiveness of context effects in our everyday decisions, scholars recently shifted their attention towards models aimed at accounting for the underlying cognitive mechanisms responsible for multiple decoy effects—that is, capable of explaining both ADDs and PDs within the same theoretical framework. For example, sequential sampling models (SSMs), such as the drift–diffusion model (Krajbich and Rangel 2011), the linear ballistic accumulator model (Trueblood et al. 2014), and the multialternative decision field theory (Roe et al. 2001), assume that the decision maker accumulates evidence for the alternatives using options’ attributes as input of a comparative process. These comparative processes affect the allocation of subjective values and are translated into choice once a certain threshold is reached (Noguchi and Stewart 2014). Indeed, the most plausible reason a non-chosen irrelevant alternative can affect a decision maker’s preference order is that the preferred alternative is previously compared with other options (Choplin and Hummel 2005). From a theoretical point of view, SSMs explain the elicitation of context effects both in value-based and perceptual choices (Busemeyer et al. 2019) and recently showed a good fit to empirical data (see Turner et al. 2018 for a comparison of the most influential models).

### Context effects in non-human animals

As mentioned above, recent findings on the merely perceptual nature of context effects suggest that they could be rooted in our evolutionary history and may have played an adaptive role in shaping our decisional mechanisms. Indeed, context effects appear to be widespread in non-human animals (Parrish et al. 2015), possibly pointing at an interspecific use of some comparative evaluation mechanisms, which results in systematic violations of basic rationality axioms. ADDs have been reported to affect choice in many non-human species, such as slime molds (Latty and Beekman 2011), starlings and hummingbirds (Bateson et al. 2002; Morgan et al. 2012), honeybees (Shafir et al. 2002) and dogs (Jackson and Roberts 2021). As regards PDs, there is only preliminary evidence supporting their effectiveness in shifting preferences in non-human animals. In the environment, phantom alternatives represent any resource that is visible and superior but unavailable at the moment of choice. For example, Tan et al. (2015) proved that empty food feeders affected honeybees’ choices. In this scenario, a bee landing on a flower only to discover it lacks nectar mirrors a situation in which a consumer realizes that the product she wants to buy is sold out (Tan et al. 2015). Scarpi (2011) reported a preference change in cats choosing between different food options in which one alternative was unreachable. Building upon the food example, an animal may encounter an unavailable alternative whenever it is unable to reach a visible or smelled food option. Lea and Ryan (2015) documented an influence of an unavailable decoy in mate choices of túngara frogs. In all three studies, adding a phantom alternative increased the selection of the most similar dominated alternative. Moreover, a recent study on free-ranging swamp wallabies (Orlando et al. 2023) reported an effect of phantom decoys mostly on information gathering behavior, since the presence of a superior yet unattainable feeding option (a food amount that could be smelled and observed, but not consumed) increased the wallabies’ inclination to examine both non-decoy alternatives before making their choice; the choice itself, however, was not significantly affected, in partial contrast with other studies. Overall, studying phantom decoys in non-human animals is crucial because it helps reveal whether different species exhibit context-dependent decision-making even when an alternative cannot be chosen, providing insights into the cognitive comparative processes underlying their choices. In fact, it has been argued that phantom decoys are very ecologically relevant, due to their frequent presence in nature, under many guises: for instance, the smell of a food item out of reach, a food item that appears rewarding but is empty (e.g., nectarless flowers encountered by foraging bees, Tan et al. 2015), or a food being consumed by a neighbor (Orlando et al. 2023).

Regarding primates, existing evidence on ADDs is more controversial and inconclusive (Table 1). A first perceptual task by Parrish et al. (2015) found an attraction effect in macaques choosing among geometric figures. However, the following study with the same species failed to replicate the effect (Parrish et al. 2018). Similar negative results have been reported in comparable studies on other primate species, such as capuchins monkeys (Cohen and Santos 2017) and great apes (Sanchez-Amaro et al. 2019), choosing between different food options. Despite this corpus of negative evidence, two recent studies on capuchin monkeys made a methodological improvement directly measuring monkeys’ preferences at baseline levels, which led to observing significant preference shifts towards the target option using ADDs (Watzek and Brosnan 2020; Marini et al. 2023). Human literature recently clarified that context effects are often muted when decision makers already have a strong preference among alternatives, prior to the introduction of an asymmetrically dominated decoy (Huber et al. 2014; Farmer et al. 2016; Gaudeul and Crosetto 2019). This suggests that a decoy is mostly effective in shifting baseline preferences when options are close to the decision maker’s indifference point. In Watzek and Brosnan’s (2020) and Marini and colleagues (2023) studies, ternary choice sets that included a decoy were built after assessing the order of preference for the two foods involved and estimating the indifference point between the less favorite and the most favorite food for each animal. These studies represented the first observations of the decoy effect in primates in a value-based task.

**Table 1:** Previous studies on decoy effects in primates

Study	Target group	Sample	Task	Results
Parrish et al. (2015)	Rhesus monkeys	*N* = *7*	Perceptual task	Attraction
Cohen and Santos (2017)	Capuchin monkeys	*N* = *7*	Value-based task	None
Parrish et al. (2018)	Rhesus monkeys	*N* = *7*	Task preference	None
Sánchez-Amaro et al. (2019)	Great apes (all species)	*N* = *32*	Value-based task	None
Watzek and Brosnan (2020)	Capuchin monkeys	*N* = *13*	Value-based task	Attraction
Marini et al. (2023)	Capuchin monkeys	*N* = *14*	Value-based task	Attraction and repulsion

Moreover, in Marini et al. (2023) study, capuchin monkeys seemed to be sensitive to the distance between the decoy and the target in the attribute space. More specifically, building the dominated decoys by halving the target amount and employing a fixed amount of the favorite food (two units) and different amounts of the non-favorite food (up to eight units) led Marini and colleagues to employ decoys with a different degree of similarity with the target options. In doing so, they reported a violation of context independence in two opposite directions. Capuchin monkeys exhibited a strong attraction effect when the decoy was very similar to the target (in the favorite food condition) and a consistent repulsion effect (i.e., an increase in competitor preferences) when the irrelevant option was farther from the dominant alternative (in the less favorite food condition). This result is not new in the human literature (Spektor et al. 2018; Evans et al. 2021), and it is coherent with a recent study that examined the impact of the distance of the decoy in the attribute space (Liao et al. 2021).

### The present study

Whereas evidence of ADDs in non-human primates is mixed, it appears to be entirely non-existent with respect to PDs: to the best of our knowledge, no study has yet investigated the effect of unavailable decoys in non-human primates. Thus, the primary objective of the present study is to fill this gap by investigating the effects of unavailable decoys (both dominated and dominating) in capuchin monkeys using an experimental protocol in which capuchins were presented with choices including, across different trials, both ADDs and PDs. We have chosen capuchin monkeys as their decision-making abilities have been widely investigated, in a comparative framework, by different authors. For instance, it has been reported that capuchins share with humans several decisional biases, as the framing effect (Chen et al. 2006) and the endowment effect (Lakshminarayanan et al. 2011). Moreover, capuchins’ sensitivity to decoys in the context of value-based choices has been recently highlighted by two independent studies (Watzek and Brosnan 2020; Marini et al. 2023), and we believe further research is necessary to illuminate aspects which are still unclear.

As in previous studies, we first estimated capuchins’ preferences in binary choices, to later administer ternary trials in which target and competitor options were near the subjective indifference points. We had three different decoy conditions, depending on the type of decoy being included: (a) a phantom decoy that dominated the favorite or the non-favorite food option (PD conditions), to investigate the influence of potentially relevant yet unavailable options on subjects’ preferences; (b) an available asymmetric decoy dominated by the favorite or the non-favorite food option (ADD conditions), to confirm its effectiveness in shifting capuchins’ preferences (Marini et al. 2023); (c) an unavailable version of the same asymmetric decoy dominated by the favorite or the non-favorite food (UADD conditions), to assess the influence of inferior unavailable decoys on capuchins’ choice behavior. This third condition amounts to introducing an inferior unavailable decoy (as it was done with honeybees in Tan et al. 2015) and it allows for direct comparison with asymmetrically dominated real decoys, since the decoys are the same in ADD and UADD conditions, except they are available in one condition, and unavailable in the other. Obviously, the same direct comparison is impossible with superior phantom decoys (those used in PD conditions), since introducing an available superior option in the choice set would inevitably result in a significant share of choices for that option, that would thus not work as a decoy.

In our experiment, we initially assessed the monkeys’ preferences and indifference points for different food items. Subsequently, we implemented a baseline phase followed by a 3X2 experimental design. This design involved the inclusion of three distinct decoy types and two directions, representing both preferred and less-preferred food targets.

### Hypotheses

This paradigm was developed to test the following hypotheses:

**H**_**1**_) *Adding a phantom decoy to a binary choice set strengthens target preferences:* our primary hypothesis assumed an impact of the unavailable superior option (phantom decoy; PD) on the choice share in both the decoy directions. Since the only way for the unavailable option (PD) to affect capuchins' preferences is to be compared with other options in the choice set, this result would represent further evidence supporting SSMs and comparative models, as well as providing the first evidence ever reported of the effectiveness of phantom decoys in non-human primates.

**H**_**2**_) *Asymmetrically dominated decoys increase target share regardless of food preferences:* we hypothesized that adding an asymmetrically dominated decoy (ADD) would affect capuchins’ preferences, increasing target selections regardless of the decoy direction (favorite or less-favorite food). In short, we expected the elicitation of the attraction effect in both ADD conditions. We supposed that previous findings on the repulsion effect were, therefore, due to an asymmetric location of the decoy in the attribute space (Liao et al. 2021; Marini et al. 2023).

**H**_**3**_) *Asymmetrically dominated decoys induce an attraction effect even when they are unavailable:* since previous literature confirmed that non-human animals are able to recognize the dominance relationship within the choice set (target-decoy), choosing decoy alternatives in a negligible number of trials, we assumed that making the dominated alternative unavailable (“unavailable asymmetrically dominated decoy”, UADD) would not interfere with the elicitation of an attraction effect. Such a result would demonstrate that unavailable decoys are able to affect capuchins’ choices also when inferior to their target option.

## Methods

### Ethical note

The study protocol was approved by the Italian Ministry of Health (337/2017-PR to EA) and fully complied with the Directive 2010/63/EU on the protection of animals used for scientific purposes.

### Subjects

A group of 12 capuchin monkeys (6 females and 6 males) with an average age of 24.6 years (ranging from 12 to 36 years) was selected for the purposes of the present study. These monkeys were housed in four different groups at the Primate Center of the Institute of Cognitive Sciences and Technologies of the National Research Council of Italy (ISTC-CNR) located in Rome. The capuchin groups were accommodated in compartments that provided both indoor and outdoor spaces. The size of the outdoor compartments varied depending on the group size, ranging from 65.4 to 139.5 m^3^. The two indoor compartments measured a total of 25.4 m^3^. Three experimental compartments (1 m^3^ in total) were attached to one of the two indoor compartments. Indoor–outdoor compartments were equipped with various enriching materials. Animals were never food deprived for testing, and they received their main meal (a variety of seasonal fruit and vegetables, along with primate monkey chow – Altromin-A, A. Rieper S.p.A., Vandoies, Italy) in the afternoon after testing was completed. Water was continuously available.

### Experimental apparatus

**Fig. 1:**
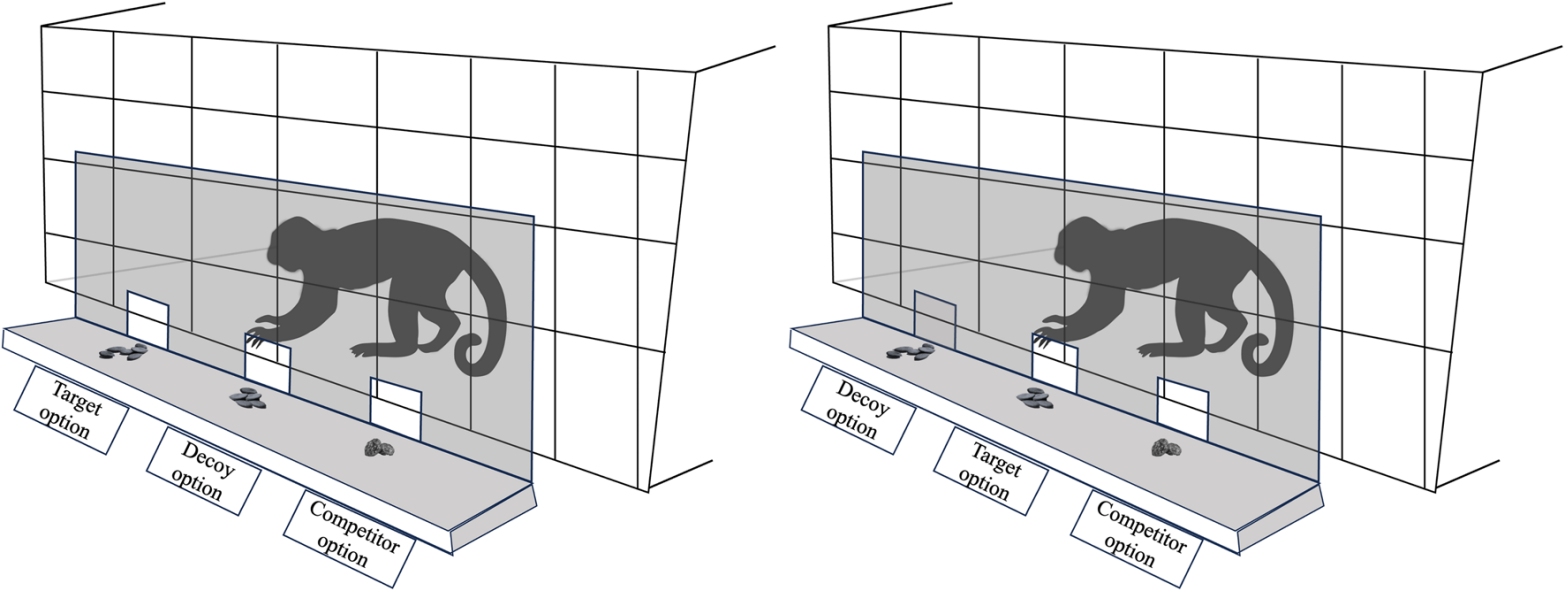
The image illustrates the apparatus utilized in the asymmetrically dominated (left) and unavailable dominating decoy (right) conditions. The options were presented using a wooden tray equipped with a vertical transparent plastic panel. The transparent panel had three openings through which the food options could be reached. In scenarios where only two options were available, one of the openings was closed off with a transparent plastic panel, allowing the food positioned behind it to remain visible

The apparatus was located on a 70.5 × 50.5 cm table (height: 87.5 cm) placed in front of the experimental compartment. In the Preliminary Food Preference phase two food options were presented by means of a Plexiglas tray (27 × 40 cm); each food option was positioned at 5.2 cm from the frontal edge of the tray and at 8 cm from a central panel that divided the tray into two equally sized portions. In all other phases, the options were presented by means of a wooden tray (43 × 50 cm) on which a vertical frame surrounding a plastic panel (50 × 40 cm) was inserted (see Fig. 1). In the Decoy phase, in “PD Unreachable” and “AD Unreachable” trials (see paragraph – *Decoy phase*), decoys were clearly visible to the experimental subject but could not be selected (a 7 × 7 cm transparent panel prevented the subject from reaching for the decoy).

### General procedure

Capuchins were tested in dedicated experimental compartments. The testing sessions took place between 09:30 a.m. and 13:00 p.m. At the beginning of the testing procedure, a subject was isolated from its social group using sliding doors, which allowed the subject to move into the experimental compartment. After the testing session, the subject immediately rejoined the social group. The study involved five experimental phases, namely Preliminary Food Preference, Pretest I, Bound Pretest, Pretest II, and Decoy Phase, which were presented in a sequential order. During the experiment, capuchins were presented with either binary or ternary choices, and they were required to select only one food option in each trial. In all phases, a first experimenter positioned the food options on the tray and pushed it toward the subject, and a second experimenter scored the data. The first experimenter prevented the subject from seeing the food options by placing a black vertical panel between the tray and the experimental subject. In order to avoid giving the subject any verbal or visual cues, both experimenters remained silent and avoided looking at any option. A 10-s intertrial interval was implemented between each trial. During the entire experimental process, video recording was conducted using a Canon Legria HF R806 camera.

### Experimental phases

#### Preliminary food preference phase

This phase aimed to identify for each subject a high-preferred familiar food (A-food) and a less-preferred familiar food (B-food), such that the A-food was chosen over the B-food in 70–80% of the trials in two consecutive sessions. Exclusively for the first session and just once, if the subject preferred one of the two foods in 65–85% of the trials, the session was repeated rather than immediately discarded. In each session, capuchins performed 20 trials of binary choices between 1 unit of food A and 1 unit of food B. The order of presentation was randomized ensuring a counterbalanced right-left placement of the options. Food was cut into pieces of approximately the same size and weight (Raisins: 0.20 g; Pumpkin seed: 0.08 g; Rice Krispies: 0.04 g; Cheerios: 0.35 g) and each food item was weighed on a digital scale (Gibertini Europe 1700; 0.1 g accuracy). Each option consisted of a single food item. The options were presented by means of a Plexiglas tray (see *Experimental apparatus section*). After the subject entered the experimental compartment and was located in its centre, the first experimenter pushed the Plexiglas tray towards the experimental compartment, so that the subject could select one of the options.

#### Pretest I phase

This phase aimed to make sure that capuchins were able to discriminate between the food quantities involved in the subsequent phases and that quadrupling the amount of the less-preferred food (B) offered to them would be sufficient to let them forsake a smaller amount of the high-preferred food (A). Each session consisted of 18 binary choices between the food pairs 2A *vs*. 1B or 2A *vs*. 8B (9 trials for each combination) presented in a pseudo-random order counterbalancing right-left position. The options were presented by means of a wooden tray (see *Experimental apparatus section*). After the subject entered the experimental compartment and located in its centre, the first experimenter pushed the wooden tray towards the experimental compartment. Monkeys moved to the next experimental phase once they demonstrated a consistent pattern of choosing 2A over 1B and 8B over 2A in at least seven out of nine trials. This criterion had to be met in two consecutive sessions to successfully complete this phase.

#### Bound pretest phase

To assess the upper bound (UB) for each monkey, which represented the minimum quantity of B-food chosen over A-food more than 50% of the time, we conducted five consecutive sessions. Each session involved 20 binary choices between 2 units of A-food and varying amounts of B-food. Within each session, subjects performed five types of trials, repeated four times, that were presented in a pseudo-random order. The right–left presentation of the options was also counterbalanced. The five types of trials included: 2A vs. 1B, 2A vs. 2B, 2A vs. 4B, 2A vs. 6B, and 2A vs. 8B. The food choices were presented using the same wooden tray of the Pretest I phase. Each subject completed a total of five sessions. This phase aimed to ensure that the decoys would be effective in influencing decision-makers' preferences, as prior research indicates that decoys are most effective when baseline option preferences are weak, but less so when there is a strong preference for one option.

#### Pretest II phase

This phase aimed to make sure that capuchins correctly perceived the dominance relationship between various types of decoys and their targets (T): for phantom decoys (PD), decoys provided the same food of the target option plus one food unit, and we expected that PD was chosen over T; for asymmetrically dominated decoys (ADD), decoys always consisted of the same type of food as their target minus one food unit, and we expected that T was chosen over ADD. Each session consisted of 36 binary choices between these options:

- 2A vs. 1A (ADD targeting high-preferred food)
- 2A vs. 3A (PD targeting high-preferred food)
- UB vs. (U-1)B (ADD targeting less-preferred food)
- UB vs. (U + 1)B (PD targeting less-preferred food)

In each session, there were nine trials of each type, presented in a pseudo-random order, counterbalancing right–left presentation. The food options were presented on the same wooden tray used in the Pretest I and in the Bound pretest phases. Pretest II was considered completed once the subject chose the target option in at least seven out of nine trials across two consecutive sessions.

#### Decoy phase

The food amounts used during the Decoy phase were chosen based on capuchins’ preferences during the Bound pretest phase aiming to be as close as possible to each subject's indifference point. In this phase, a 3 × 2 within-subject design was employed (three decoy types: phantom decoys, PD; unavailable asymmetrically dominated decoys, UADD; asymmetrically dominated decoys, ADD; two target food types: High Preferred, A-food; Less Preferred, B-food). Thus, there were six experimental conditions: PD on high preferred, PD on less preferred, UADD on high preferred, UADD on less preferred, ADD on high preferred, ADD on less preferred. Within each session, subjects were presented with 4 trials of each type, plus 4 baseline binary choices 2A vs. UB (where U is the upper bound for each subject), resulting in a total of 28 trials per session. The list of conditions is provided in Table 2.

**Table 2:** List of experimental conditions

Name	Conditions			Options		
	Choice	Decoy type	Target option	High Preferred food amount	Less preferred food amount	Decoy
Baseline	Binary	n.a	n.a	2A	UB	–
PD—HPF	Ternary	Phantom, superior	High-preferred food	2A	UB	3A
PD—LPF	Ternary	Phantom, superior	Less-preferred food	2A	UB	UB + 1
UADD—HPF	Ternary	Unavailable, asymmetrically dominated	High-preferred food	2A	UB	1A
UADD—LPF	Ternary	Unavailable, asymmetrically dominated	Less-preferred food	2A	UB	UB-1
ADD—HPF	Ternary	Available, asymmetrically dominated	High-preferred food	2A	UB	1A
ADD—LPF	Ternary	Available, asymmetrically dominated	Less-preferred food	2A	UB	UB-1

Baseline (2 units of the preferred food) and UB (the threshold at which the monkeys consistently displayed a preference for the B-food option over the A-food option in the majority of their choices) were always available. In ternary conditions we added a decoy as either a superior (unavailable) or inferior (available or unavailable) option to the choice set

Trial presentation was pseudo-randomized within each session, ensuring that two consecutive trials were never of the same type. Decoy placement (left, center, right) was also pseudo-randomized within the four trials of the same type in any given session, making sure decoy and target were always next to each other in ternary trials. Decoys were designed as in Pretest II: PD consisted of the same type of food as their target, and in the same amount plus one food item; ADD consisted of the same type of food as their target, and in the same amount minus one food item. Both decoys were designed in such a way as to be asymmetrically dominated by (ADD) or dominant over (PD) their target, but never their competitor.

In PD and UADD trials, decoys were clearly visible to the subject but could not be selected (a 7 × 7 cm transparent panel prevented the animal from reaching for the decoy; see *Experimental apparatus section*); in ADD trials, in contrast, decoys were accessible and could be chosen. Target and competitor were always accessible to the subject, across all trials. The food options were presented using the wooden tray used in previous phases.

### Data analysis

Only the data for the Decoy phase were statistically analysed by means of the software Stata IC (Version 14). The dependent variable was the choice for the 2A option (namely, two units of the favourite food). To control for individuals’ variability, subjects’ identity was modeled as a random effect, sex and condition as categorical predictors, and age, session number and trial number as continuous predictors. Conditional fixed-effects logistic regressions with robust standard errors were employed and the significance of interaction effects was tested using the Wald test.

## Results

### Preliminary food preference phase

In this phase, a food pair was chosen for each subject. This food pair was selected ensuring that A-food was preferred over B-food in 65–85% of the trials in two consecutive sessions. All monkeys showed consistent preferences, with an average of 77% of choices favoring the A-food. The food pairs employed for each animal can be found in Table S1 in the Appendix.

### Pretest I phase

All subjects preferred two pieces of the high preferred A-food over one piece of the less preferred B-food and eight pieces of the B-food over two pieces of the A-food. Ten subjects reached the criterion of choosing 2A over 1B and 8B over 2A in at least 7 trials out of 9 in two sessions and two subjects (Quincy and Patè) in three sessions (Appendix, Table S2).

### Bound pretest phase

Table 3 reports the results of the Bound pretest phase, which aimed to find for each subject an amount of B-food defined UB (upper bound), such that it was the smallest quantity of B-food that the subject chose in more than 50% of the trials over the A-food.

**Table 3:** Bound pretest phase

Subject	Preferred food	Upper bound	2A vs. UB
Patè	2A	4B	0.20
Penelope	2A	6B	0.25
Peonia	2A	4B	0.25
Pepe	2A	6B	0.30
Quincy	2A	4B	0.40
Roberta	2A	4B	0.30
Robinia	2A	4B	0.45
Robot	2A	4B	0.45
Sandokan	2A	6B	0.20
Saroma	2A	4B	0.00
Totò	2A	4B	0.35
Vispo	2A	4B	0.35

For each subject, upper bound (number of B-food units) and proportion of preferences for 2A in the comparisons 2A vs. UB

### Pretest II phase

In the Pretest II phase, all subjects successfully chose the target option over the decoy option in at least seven out of nine trials. Nine subjects reached the criterion in two sessions, two subjects (Penelope and Pepe) in three sessions and one subject (Quincy) needed five sessions. Preferences are reported in Table S3 in the Appendix.

### Decoy phase

Descriptive statistics are reported in Table 4.

**Table 4:** Decoy phase. For each experimental subject, proportion of 2A preferences in each experimental condition

Subject	Baseline	PD on A	PD on B	UADD on A	UADD on B	ADD on A	ADD on B
	2A/UB	2A/1A/UB	2A/UB-1/UB	2A/3A(u)/UB	2A/UB + 1(u)/UB	2A/1A(u)/UB	2A/UB-1(u)/UB
Patè	0.12	0.55	0.05	0.45	0.02	0.20	0.15
Penelope	0.82	0.90	0.67	0.87	0.60	0.80	0.47
Peonia	0.25	0.80	0.25	0.52	0.17	0.65	0.22
Pepe	0.82	0.85	0.57	0.90	0.52	0.65	0.65
Quincy	0.50	0.85	0.27	0.70	0.42	0.67	0.27
Roberta	0.62	0.82	0.17	0.75	0.17	0.77	0.35
Robinia	0.72	0.90	0.07	0.97	0.30	0.90	0.12
Robot	0.42	0.80	0.05	0.75	0.02	0.57	0.05
Sandokan	0.87	0.92	0.50	0.90	0.27	0.87	0.45
Saroma	0.67	0.97	0.27	0.80	0.22	0.70	0.17
Totò	0.65	0.85	0.27	0.82	0.15	0.82	0.20
Vispo	0.44	0.80	0.02	0.75	0.10	0.62	0.07

**Fig. 2:**
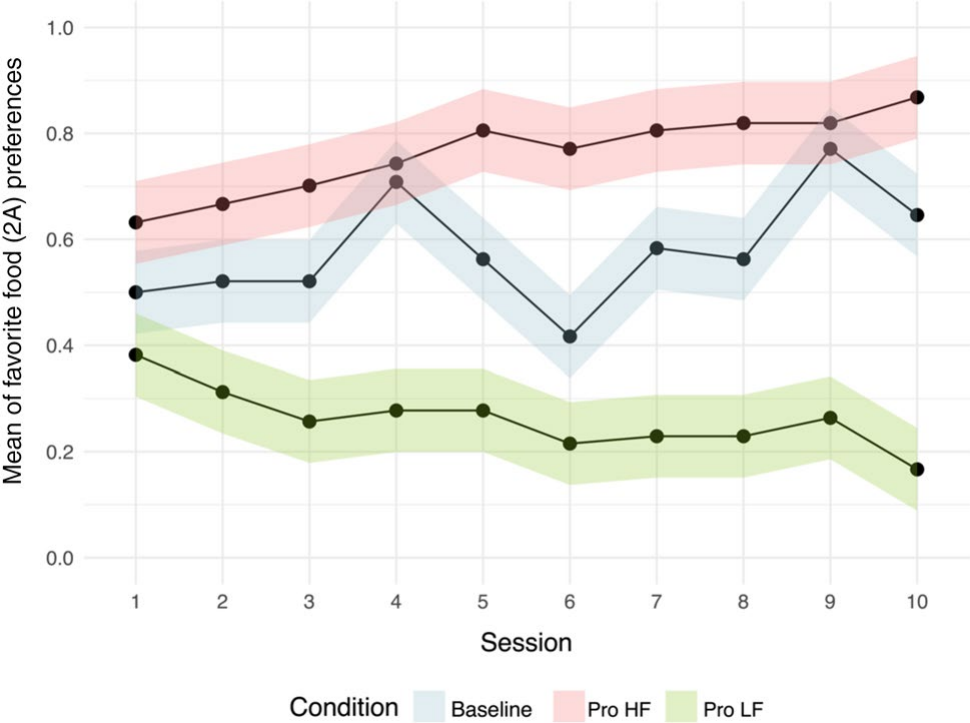
Capuchins’ proportion of choices for the option 2A (favourite food) as a function of the experimental session. In High-preferred food and Less-preferred food conditions, target food preferences (favourite and non-favourite food) increased over time. Error bars indicate 95% confidence intervals

Conditional fixed-effects logistic regressions with robust standard errors were employed to evaluate the influence of session, trial number, and condition (fixed effects) on the target preferences (dependent variable). The participants' intercepts and slopes have been incorporated as random effects. There was a significant interaction between condition and session (χ^2^_6_ = 318.81; *p* < 0.001), as shown in Fig. 2. In High-preferred food conditions (*N* = 480 trials), the choices for the 2A option significantly increased across sessions in the ADD (*z* = 4.00, coeff. = 0.184, *p* < 0.001) and UADD conditions (*z* = 2.15, coeff. = 0.150, *p* = 0.032), and showed a positive but non-significant trend in the PD condition (*z* = 1.79, coeff. = 0.108, *p* = 0.073). In Less-preferred food conditions (*N* = 480 trials), the choices for the 2A option significantly decreased across sessions in the UADD (*z* = − 2.24, coeff. = − 0.113, *p* = 0.025) and PD conditions (*z* = − 2.37, coeff. = − 0.101, *p* = 0.018), whereas for the AD condition there was a non-significant decrease (*z* = -1.26, coeff. = − 0.057, *p* = 0.207; see Fig. 1). There was also a significant interaction between condition and trial (χ^2^_6_ = 16.93; *p* < 0.01), but *post hoc* analysis did not show any significant variation in 2A choices over trials within the same session (for complete details of the statistical outputs, please see the Appendix).

**Fig. 3:**
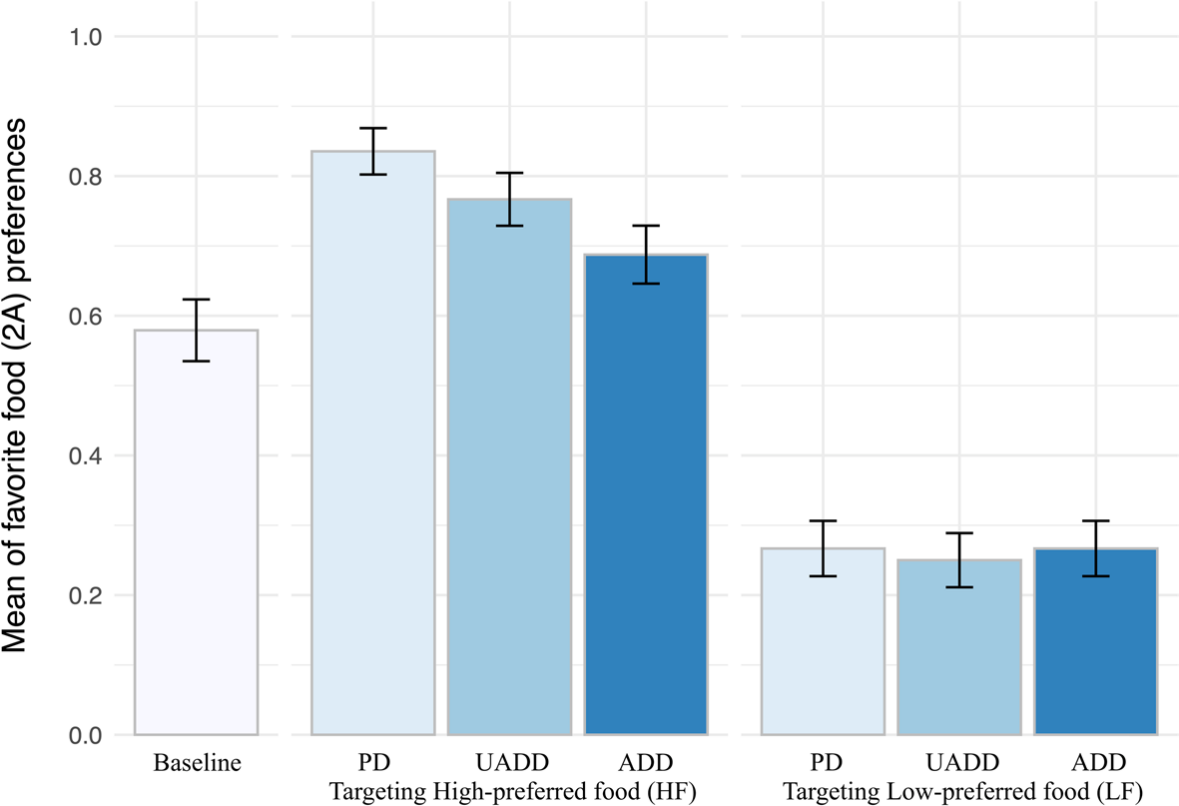
Attraction and unavailable decoy effect. Capuchins’ proportion of choices for the option 2A (favourite food) as a function of the experimental conditions. Error bars indicate 95% confidence intervals

As shown in Fig. 3, capuchins chose the 2A option more frequently in all High-preferred food conditions than in the Baseline. After Bonferroni correction, which lowered the alpha level to 0.0042 (alpha = 0.05/12 comparisons), this difference resulted significant for the comparisons UADD vs. Baseline (*z* = 6.83, coeff. = 0.961, *p* < 0.001) and PD *vs*. Baseline (*z* = 8.31, coeff. = 1.473, *p* < 0.001), but not for the comparison ADD vs. Baseline (*z* = 2.75, coeff. = 0.529, *p* = 0.006). Conversely, capuchins chose the 2A option significantly less frequently in all Less-preferred food conditions than in the Baseline (ADD vs. Baseline: *z* = − 6.08, coeff. = − 1.494, *p* < 0.001; UADD vs. Baseline: *z* = − 6.41, coeff. = − 1.631, *p* < 0.001; PD vs. Baseline: *z* = 5.95, coeff. = 1.507, *p* < 0.001; Fig. 3). Moreover, in High-preferred food conditions, capuchins chose the 2A option significantly more frequently in PD than in ADD trials (*z* = 6.00, coeff. = 0.944, *p* < 0.001) and in PD than in UADD trials (*z* = 3.07, coeff. = 0.512, *p* = 0.002), but not in UADD than in ADD trials (*z* = 2.34, coeff. = 0.431, *p* = 0.020; Bonferroni corrected alpha = 0.0042). From pairwise comparisons of the Less-preferred food conditions, no significant difference emerged, as can be seen in Fig. 3. For complete details of the statistical outputs, please see the Appendix.

## Discussion

Our results confirm all three hypotheses of this study: firstly, unavailable decoys influenced the preferences of capuchin monkeys, both when they were superior to their target (PD conditions) and when they were instead dominated by it (UADD conditions). This result provides the first experimental evidence of phantom decoy effects in non-human primates available in the literature. Secondly, asymmetrically dominated decoys were effective in shifting capuchins’ preferences regardless of their target (whether high-preferred or less-preferred food), thus suggesting that previous anomalies in the effectiveness of ADDs (i.e., repulsion effects; Marini et al. 2023) were likely due to the different positioning of the decoy in the attribute space (Liao et al. 2021). Lastly, ADDs remained effective even when they could not be selected by capuchins (UADD conditions), thus suggesting that their main role in the decision-making process responsible for the attraction effect is comparative and, therefore, unaffected by actual availability. In short, all three types of decoys used in this study increase preferences for the target food in the ternary conditions, regardless of whether the target was the high-preferred or less-preferred food.

More generally, the specific design of this study allowed us to demonstrate that making the decoys unavailable does not diminish their impact on capuchins’ preferences: this remains true both for unavailable options that are superior to their target (PDs) and for those that are asymmetrically dominated by it (UADDs). Indeed, the influence of unavailable decoys on choice was indistinguishable from that of available decoys in Less-preferred food conditions, and even slightly stronger in High-preferred food conditions. Such a significant role of unavailable options in decision making is consistent with sequential sampling models (SSMs), according to which decision makers gain information on the alternatives by using the attributes of each option as input in a comparative process. Making an option unavailable does not make its attributes any less accessible as information input for the sake of comparison: in fact, making decoys unobtainable in the choice set renders them irrelevant as consumable options and this might force subjects to regard them more as a yardstick, therefore, enhancing the salience of the relevant dominance relationship with their target (which would explain why the effects appear to be no weaker, and are occasionally stronger, than those elicited by available decoys). Further studies will be needed to investigate the specific factors responsible for the effectiveness of unavailable options as decoys. Nonetheless, the data collected in this study, and in particular the comparison between ADD and UADD conditions, provides a first valuable set of evidence on the relative effectiveness of available and unavailable decoys.

With respect to the preexisting literature on decoy effects in non-human primates, this study offers two notable indications:

- It provides the first ever evidence of the effectiveness of unavailable decoys in influencing choices in non-human primates in ways similar to humans, and it documents such effects across a significant variety of conditions: using both superior and inferior phantom decoys, and targeting both high-preferred and less-preferred foods. We are confident that this will invite further research on dominating and dominated unavailable decoys in other primate species, besides capuchin monkeys.
- It confirms that (available or unavailable) asymmetrically dominated decoys can shift preferences towards their target in non-human primates, consistently with the most recent studies (Watzek and Brosnan 2020; Marini et al. 2023) and in contrast to prior attempts (Cohen and Santos 2017; Sanchez-Amaro et al. 2019). Here, the fact that only studies who pre-assessed binary preferences at the individual level for each animal were successful in eliciting decoy effect in value-based choice tasks (as opposed to perceptual discrimination tasks, as used in Parrish et al. 2015, 2018) strongly suggests that such practice should become standard in this type of study; this is also consistent with what we know of decoy effects in human subjects (Huber et al. 2014; Farmer et al. 2016; Gaudeul and Crosetto 2019), thus hinting at a further similarity in the decision processes responsible for this phenomenon across different species.

From a methodological standpoint, the symmetry observed for context effects across all conditions, regardless of the type of food being targeted by decoys, vindicate the soundness of the paradigm adopted in this study. In particular, it suggests that three aspects of this methodology should become a gold standard for future research on decoy effects in non-human animals: (i) the indifference point of each animal should be estimated in binary trials (see also Watzek and Brosnan 2020; Marini et al. 2023), so that individual preferences between baseline options are malleable enough to allow observing decoy effects in either direction; (ii) the distance between the decoy and the target must be carefully controlled, because it is known that different manipulations of this parameter can elicit different context effects, i.e. attraction vs repulsion (Liao et al. 2021); (iii) baseline trials should be included also within decoy sessions, to guarantee that context effects are not biased by independent shifts in baseline preferences across sessions. Taking care of these methodological constraints lead to rather elaborate experimental protocols, like the one employed in this study. Yet, we believe it is crucial to guarantee sound results, while we continue exploring context effects and what they reveal on the comparative nature of decision-making processes across various species.

## Supplementary Information

Below is the link to the electronic supplementary material.

Supplementary file1 (DOCX 24 KB)

## Data Availability

The data that support the findings of this study are openly available in OSF at: https://osf.io/pg3zx/?view_only=ac0928fbdb974c56be965405cbb9f964

## References

[CR1] Ariely D, Jones S (2008) Predictably irrational. Harper Collins, New York, pp 278–279

[CR2] Bateson M, Healy SD, Hurly TA (2002) Irrational choices in hummingbird foraging behaviour. Anim Behav 63(3):587–596

[CR3] Bordalo P, Gennaioli N, Shleifer A (2013) Salience and consumer choice. J Polit Econ 121(5):803–843

[CR4] Busemeyer JR, Gluth S, Rieskamp J, Turner BM (2019) Cognitive and neural bases of multi-attribute, multi-alternative, value-based decisions. Trends Cogn Sci 23(3):251–263 30630672 10.1016/j.tics.2018.12.003

[CR5] Chen MK, Lakshminarayanan V, Santos LR (2006) How basic are behavioral biases? evidence from capuchin monkey trading behavior. J Polit Econ 114(3):517–537

[CR6] Choplin JM, Hummel JE (2005) Comparison-induced decoy effects. Mem Cognit 33:332–343 16028587 10.3758/bf03195321

[CR7] Cohen PM, Santos LR (2017) Capuchins (*Cebus apella*) fail to show an asymmetric dominance effect. Anim Cogn 20:331–345 27853864 10.1007/s10071-016-1055-5

[CR8] Dimara E, Bailly G, Bezerianos A, Franconeri S (2018) Mitigating the attraction effect with visualizations. IEEE Trans Visual Comput Graph 25(1):850–860 10.1109/TVCG.2018.2865233 30137000

[CR9] Doyle JR, O’Connor DJ, Reynolds GM, Bottomley PA (1999) The robustness of the asymmetrically dominated effect: buying frames, phantom alternatives, and in-store purchases. Psychol Mark 16(3):225–243

[CR10] Evans NJ, Holmes WR, Dasari A, Trueblood JS (2021) The impact of presentation order on attraction and repulsion effects in decision-making. Decision 8(1):36–54

[CR11] Farmer GD, Warren PA, El-Deredy W, Howes A (2017) The effect of expected value on attraction effect preference reversals. J Behav Decis Mak 30(4):785–793 29081595 10.1002/bdm.2001 PMC5637901

[CR12] Gaudeul, A., & Crosetto, P. (2019). Fast then slow: a choice process explanation for the attraction effect. hal-02408719f

[CR13] Hedgcock W, Rao AR (2009) Trade-off aversion as an explanation for the attraction effect: a functional magnetic resonance imaging study. J Mark Res 46(1):1–13

[CR14] Herne K (1997) Decoy alternatives in policy choices: asymmetric domination and compromise effects. Eur J Polit Econ 13(3):575–589

[CR15] Huber J, Payne JW, Puto C (1982) Adding asymmetrically dominated alternatives: violations of regularity and the similarity hypothesis. J Consumer Res 9(1):90–98

[CR16] Huber J, Payne JW, Puto CP (2014) Let’s be honest about the attraction effect. J Mark Res 51(4):520–525

[CR17] Jackson SM, Roberts WA (2021) Irrational behavior in dogs (Canis lupus familiaris): a violation of independence from irrelevant alternatives. Behav Proc 193:104512 10.1016/j.beproc.2021.104512 34582936

[CR18] Kahneman D (1979) Prospect theory: an analysis of decisions under risk. Econometrica 47:263–292

[CR19] Kelman M, Rottenstreich Y, Tversky A (1996) Context-dependence in legal decision making. J Leg Stud 25(2):287–318

[CR20] Krajbich I, Rangel A (2011) Multialternative drift-diffusion model predicts the relationship between visual fixations and choice in value-based decisions. Proc Natl Acad Sci 108(33):13852–13857 21808009 10.1073/pnas.1101328108 PMC3158210

[CR21] Lakshminarayanan VR, Chen MK, Santos LR (2011) The evolution of decision-making under risk: framing effects in monkey risk preferences. J Exp Soc Psychol 47(3):689–693

[CR22] Latty T, Beekman M (2011) Irrational decision-making in an amoeboid organism: transitivity and context-dependent preferences. Proc R Soc b Biol Sci 278(1703):307–312 10.1098/rspb.2010.1045 PMC3013386 20702460

[CR23] Lea AM, Ryan MJ (2015) Irrationality in mate choice revealed by túngara frogs. Science 349(6251):964–966 26315434 10.1126/science.aab2012

[CR24] Liao J, Chen Y, Lin W, Mo L (2021) The influence of distance between decoy and target on context effect: attraction or repulsion? J Behav Decis Mak 34(3):432–447

[CR25] Lichtenstein S, Slovic P (eds) (2006) The construction of preference. Cambridge University Press, Cambridge

[CR26] Lichters M, Sarstedt M, Vogt B (2015) On the practical relevance of the attraction effect: a cautionary note and guidelines for context effect experiments. AMS Rev 5(1):1–19

[CR27] Marini M, Paglieri F (2019) Decoy effects in intertemporal and probabilistic choices the role of time pressure, immediacy, and certainty. Behav Proc 162:130–141 10.1016/j.beproc.2019.03.002 30849515

[CR28] Marini M, Ansani A, Paglieri F (2020) Attraction comes from many sources: attentional and comparative processes in decoy effects. Judgm Decis Mak 15(5):704–726

[CR29] Marini M, Sapienza A, Paglieri F (2022) There is more to attraction than meets the eye: studying decoy-induced attention allocation without eye tracking. J Behav Decis Mak 2023(36):e2299

[CR30] Marini M, Boschetti C, Gastaldi S, Addessi E, Paglieri F (2023) Context-effect bias in capuchin monkeys (*Sapajus* spp.): exploring decoy influences in a value-based food choice task. Anim Cognit 503–514 10.1007/s10071-022-01670-0 PMC9950244 36125642

[CR31] Morgan KV, Hurly TA, Bateson M, Asher L, Healy SD (2012) Context-dependent decisions among options varying in a single dimension. Behav Proc 89(2):115–120 10.1016/j.beproc.2011.08.017 21945144

[CR32] Noguchi T, Stewart N (2014) In the attraction, compromise, and similarity effects, alternatives are repeatedly compared in pairs on single dimensions. Cognition 132(1):44–56 24762922 10.1016/j.cognition.2014.03.006

[CR33] Orlando CG, Banks PB, Latty T, McArthur C (2023) To eat, or not to eat: a phantom decoy affects information-gathering behavior by a free-ranging mammalian herbivore. Behav Ecol 34(5):759–768 37744169 10.1093/beheco/arad057 PMC10516680

[CR34] Parrish AE, Evans TA, Beran MJ (2015) Rhesus macaques (*Macaca mulatta*) exhibit the decoy effect in a perceptual discrimination task. Atten Percept Psychophys 77:1715–1725 25832189 10.3758/s13414-015-0885-6 PMC4470728

[CR35] Parrish AE, Afrifa E, Beran MJ (2018) Exploring decoy effects on computerized task preferences in rhesus monkeys (*Macaca mulatta*). Anim Behav Cognit 5(2):235–253

[CR36] Pettibone JC, Wedell DH (2000) Examining models of nondominated decoy effects across judgment and choice. Organ Behav Hum Decis Process 81(2):300–328 10706818 10.1006/obhd.1999.2880

[CR37] Pettibone JC, Wedell DH (2007) Testing alternative explanations of phantom decoy effects. J Behav Decis Mak 20(3):323–341

[CR38] Pratkanis AR, Farquhar PH (1992) A brief history of research on phantom alternatives: evidence for seven empirical generalizations about phantoms. Basic Appl Soc Psychol 13(1):103–122

[CR39] Prelec D, Wernerfelt B, Zettelmeyer F (1997) The role of inference in context effects: inferring what you want from what is available. J Consumer Res 24(1):118–125

[CR40] Rieskamp J, Busemeyer JR, Mellers BA (2006) Extending the bounds of rationality: evidence and theories of preferential choice. J Econ Lit 44(3):631–661

[CR41] Roe RM, Busemeyer JR, Townsend JT (2001) Multialternative decision field theory: a dynamic connectionst model of decision making. Psychol Rev 108(2):370–392 11381834 10.1037/0033-295x.108.2.370

[CR42] Sánchez-Amaro A, Altinok N, Heintz C, Call J (2019) Disentangling great apes’ decoy-effect bias in a food choice task. Anim Behav Cognit 6(3):213–222

[CR43] Scarpi D (2011) The impact of phantom decoys on choices in cats. Anim Cogn 14:127–136 20838836 10.1007/s10071-010-0350-9

[CR44] Scarpi D, Pizzi G (2013) The impact of phantom decoys on choices and perceptions. J Behav Decis Mak 26(5):451–461

[CR45] Schwartz JA, Chapman GB (1999) Are more options always better? the attraction effect in physicians’ decisions about medications. Med Decis Making 19(3):315–323 10424838 10.1177/0272989X9901900310

[CR46] Shafir S, Waite TA, Smith BH (2002) Context-dependent violations of rational choice in honeybees (*Apis mellifera*) and gray jays (*Perisoreus canadensis*). Behav Ecol Sociobiol 51:180–187

[CR47] Simonson I (1989) Choice based on reasons: the case of attraction and compromise effects. J Consumer Res 16(2):158–174

[CR48] Spektor MS, Kellen D, Hotaling JM (2018) When the good looks bad: an experimental exploration of the repulsion effect. Psychol Sci 29(8):1309–1320 29792774 10.1177/0956797618779041

[CR49] Tan K, Dong S, Liu X, Chen W, Wang Y, Oldroyd BP, Latty T (2015) Phantom alternatives influence food preferences in the east-ern honeybee *Apis cerana*. J Anim Ecol 84:509–517 25251672 10.1111/1365-2656.12288

[CR50] Trueblood JS, Brown SD, Heathcote A, Busemeyer JR (2013) Not just for consumers: context effects are fundamental to decision making. Psychol Sci 24(6):901–908 23610134 10.1177/0956797612464241

[CR51] Trueblood JS, Brown SD, Heathcote A (2014) The multiattribute linear ballistic accumulator model of context effects in multialternative choice. Psychol Rev 121(2):179–205 24730597 10.1037/a0036137

[CR52] Turner BM, Schley DR, Muller C, Tsetsos K (2018) Competing theories of multialternative, multiattribute preferential choice. Psychol Rev 125(3):329 29265855 10.1037/rev0000089

[CR53] Tversky A, Simonson I (1993) Context-dependent preferences. Manage Sci 39(10):1179–1189

[CR54] Watzek J, Brosnan S (2020) Capuchin monkeys (*Sapajus [Cebus] apella*) are more susceptible to contrast than to decoy and social context effects

[CR55] Wu S, Yu R (2020) The impact of phantom decoys on the neural processing of valuation. Brain Struct Funct 225:1523–1535 32385518 10.1007/s00429-020-02079-6

[CR56] Zhen S, Yu R (2016) The development of the asymmetrically dominated decoy effect in young children. Sci Rep 6(1):22678 26935899 10.1038/srep22678 PMC4776153

