# Supplementary material for: Available and unavailable decoys in capuchin monkeys (Sapajus spp.) decision-making
Source: Anim Cogn. 2024 Feb 23;27(1):3. doi: 10.1007/s10071-024-01860-y (PMC10884124; doi:10.1007/s10071-024-01860-y)
Supplement: Supplementary file 1 — Supplementary file1 (DOCX 24 KB) [file 10071_2024_1860_MOESM1_ESM.docx]

**Appendix**

| **Subject** | **A Food** | **B Food** | **% Preference A Food over B Food** | |
| --- | --- | --- | --- | --- |
|  |  |  | **Session I** | **Session II** |
| Patè | Cheerios | Pumpkin Seed | 0.80 | 0.85 |
| Penelope | Cheerios | Raisins | 0.80 | 0.80 |
| Peonia | Raisins | Rice Krispies | 0.75 | 0.80 |
| Pepe | Cheerios | Raisins | 0.75 | 0.70 |
| Quincy | Raisins | Pumpkin Seed | 0.80 | 0.75 |
| Roberta | Raisins | Rice Krispies | 0.75 | 0.75 |
| Robinia | Raisins | Pumpkin Seed | 0.75 | 0.75 |
| Robot | Raisins | Rice Krispies | 0.75 | 0.75 |
| Sandokan | Cheerios | Raisins | 0.80 | 0.80 |
| Saroma | Raisins | Rice Krispies | 0.75 | 0.75 |
| Totò | Raisins | Rice Krispies | 0.80 | 0.80 |
| Vispo | Raisins | Rice Krispies | 0.75 | 0.80 |

***Table S1*.** Preliminary Food Preference phase. For each subject, foods used and relative preferences.

| **Subject** | **Sessions** | **2A *vs*. 1B** | **2A *vs*. 8B** |
| --- | --- | --- | --- |
| Penelope | 2 | 0.94 | 0.00 |
| Peonia | 2 | 0. 94 | 0.11 |
| Quincy | 3 | 0.93 | 0.26 |
| Patè | 3 | 0.74 | 0.11 |
| Pepe | 2 | 0.89 | 0.17 |
| Robot | 2 | 1.00 | 0.06 |
| Robinia | 2 | 0.94 | 0.00 |
| Saroma | 2 | 0.89 | 0.00 |
| Sandokan | 2 | 1.00 | 0.11 |
| Vispo | 2 | 1.00 | 0.06 |
| Roberta | 2 | 1.00 | 0.11 |
| Totò | 2 | 1.00 | 0.11 |

***Table S2*.** Pre-test I phase. Number of sessions required to reach the criterion and food preferences.

| **Subject** | **Sessions** | **1A *vs*. 2A** | **2A *vs*. 3A** | **UB *vs*. UB-1** | **UB *vs*. UB+1** |
| --- | --- | --- | --- | --- | --- |
| Penelope | 3 | 0.88 | 0.33 | 0.81 | 0.11 |
| Peonia | 2 | 1.00 | 0.16 | 0.78 | 0.05 |
| Quincy | 5 | 0.84 | 0.16 | 0.81 | 0.07 |
| Patè | 2 | 0.94 | 0.11 | 1.00 | 0.00 |
| Pepe | 3 | 0.96 | 0.11 | 0.81 | 0.04 |
| Robot | 2 | 1.00 | 0.00 | 0.83 | 0.05 |
| Robinia | 2 | 1.00 | 0.05 | 0.83 | 0.11 |
| Saroma | 2 | 1.00 | 0.00 | 0.89 | 0.00 |
| Sandokan | 2 | 1.00 | 0.11 | 0.94 | 0.11 |
| Vispo | 2 | 1.00 | 0.11 | 0.89 | 0.05 |
| Roberta | 2 | 1.00 | 0.05 | 0.89 | 0.17 |
| Totò | 2 | 1.00 | 0.05 | 1.00 | 0.11 |

***Table S3*.** Pre-test II phase. Number of sessions required to reach the criterion and proportion of choices for target options (2A and UB)
